# Expression of Concern: Short-term early exposure to thirdhand cigarette smoke increases lung cancer incidence in mice

**DOI:** 10.1042/CS20171521_EOC

**Published:** 2024-11-26

**Keywords:** cigarette smoke, DNA double strand breaks, lung carcinogenesis, thirdhand smoke

The Editorial Office has been made aware of potential issues surrounding the scientific validity of this paper “Short-term early exposure to thirdhand cigarette smoke increases lung cancer incidence in mice” DOI: 10.1042/CS20171521, hence has issued an expression of concern to notify readers whilst investigations are ongoing. It has been noted by a reader that:

- Figure 3C p-p53 panel seems to appear in part as Figure 6 CUL4A panel from Wang et al, 2017 (DOI: 10.3892/ijmm.2017.3118) [retracted], and Figure 3e HOXA7 panel from Tang et al, 2016 (DOI: 10.1186/s12943-016-0540-4) [retracted] with horizontal flip transformation
- Figure 5C Control panel seems to appear as both Figure 2D shWDR5 #1 panel with contrast alteration, and Figure 2D shWDR5 #2 panel with contrast alteration and rotation from Dai et al, 2020 (DOI: 10.2147/cmar.s237582)
- Figure 5C THS panel seems to appear as Figure 2D Mock panel with contrast alteration from Dai et al, 2020 (DOI: 10.2147/cmar.s237582).

